# Theoretical Predictions of the Structural and Mechanical Properties of Tungsten–Rare Earth Element Alloys

**DOI:** 10.3390/ma14113046

**Published:** 2021-06-03

**Authors:** Mingyu Wu, Zhihang Wang, Ningning Zhang, Changchun Ge, Yujuan Zhang

**Affiliations:** Institute of Nuclear Materials, School of Materials Science and Engineering, University of Science and Technology Beijing (USTB), Beijing 100083, China; wumingyustudy@163.com (M.W.); wangzhihangustb@163.com (Z.W.); zhangningning.1211@163.com (N.Z.)

**Keywords:** tungsten, rare earth element, mechanical property, first-principles calculation

## Abstract

Tungsten (W) is considered as the potential plasma facing material of the divertor and the first wall material in fusion. To further improve the ductility of W, the structural and mechanical properties of W–M (M = rare earth element Y, La, Ce and Lu) alloys are systematically investigated by first-principles calculations. Our results reveal that all the W_1*−x*_M*_x_* (*x* = 0.0625, 0.125, 0.1875, 0.25) alloys can form binary solid solution at the atomic level, and the alloys keep bcc lattice structures until the concentration of M increases to a certain value. Although the moduli of the alloys are reduced compared to that of pure W metal, the characteristic *B/G* ratio and Poisson’s ratio significantly increase, implying all the four rare earth elements can efficiently improve the ductility of W metal. Considering both factors of mechanical strength and ductility, La and Ce are better alloying elements than Y and Lu.

## 1. Introduction

Plasma facing materials (PFMs) play an important role in effective controlling of impurities entering the plasma, transferring the heat radiated to the surface of the materials and protecting other components from being damaged by plasma bombardment during abnormal shutdown in tokamak [1]. Therefore, the PFMs of fusion reactors need to meet the stringent requirements of good compatibility with plasma, high heat load resistance, high flux energy ion and neutral particle irradiation [2,3]. Due to its advantages such as high melting point, excellent resistance to gamma radiation, high thermal conductivity, low tritium inventory and low thermal expansion coefficient, tungsten (W) has been considered as a potential PFM of the divertor [4,5]. Nevertheless, the application of W is still limited by the shortcomings of low ductility, poor fracture toughness, and high ductile–brittle transition temperature (DBTT). Thus, science researchers in the nuclear field have made great efforts to improve these weaknesses of W, and a lot of progress have been achieved in recent years [6,7,8,9,10,11].

To date, alloying with metal elements has been proved to be an effective approach to enhance the overall mechanical properties of W. Such strategies are well-accepted for alloying with Ti [4], Ni [12], Cr [13] and Zr [14,15] to improve the ductility properties of W. For example, the positive effects of Ti-alloying in W on the grain growth behaviors and fundamental mechanical properties are reported. The addition of Ti can well improve the elastic property W metal [4]. Element Zr can be easy to dissolve in W predicted by first-principles simulations, and the ductility of W–Zr alloys is improved when the content of Zr is gradually increased [14,15]. Very recently, Jiang et al. reported that W_14_Re_2_ alloy exhibits both higher mechanical strength and better ductility than pure W metal [16].

Alloying with rare earth elements to optimize the performances of W material is also becoming popular among various alloying strategies [7,17,18,19,20]. Previous works have confirmed that rare earth elements offer high chemical affinity for oxidation in W. For example, the rare earth oxides of La_2_O_3_, Y_2_O_3_ and CeO_2_ are formed when the elements of La, Y or Ce are added in W [7,11,18,19,20]. The formed oxide dispersion strengthened (ODS) phase can inhibit the grain boundary sliding to improve the ductility and reduce the DBTT. Jiang et al. [7] reported the alloying of Y metal can also largely enhance the ductility and the deformation resistance of W predicated by first-principles simulations.

However, up to now, there still have not been systematic studies to investigate the mechanical properties of tungsten-rare earth element alloys from the atomic scale. To better understand the role that the rare earth elements play in improving the performances of W material and preliminarily determine which element is better from the comprehensive view, in this paper, the structures and mechanical properties of W–M alloys (M = Y, La, Ce and Lu) have been systematically studied by first-principles calculations. The lattice constants, phase stability and elastic constants of the W_1*−x*_M*_x_* (*x* = 0.0625, 0.1250, 0.1875, 0.2500) alloys are calculated. The derived bulk modulus (*B*), shear modulus (*G*), Young’s modulus (*E*), Poisson’s ratio (*ν*) and Cauchy pressure (*C*′) of W–M alloy are given and discussed. Furthermore, the effects of the Y, La, Ce and Lu concentrations on the fundamental mechanical properties of W are also presented. We expect that the calculated data can provide a scientific basis for the experiments of W alloying with rare earth elements.

## 2. Computational Methods

The first-principles calculations were performed within the density functional theory (DFT) and the plane-wave pseudopotential method, which were implemented in the Vienna Ab-initio Simulation Package (VASP) [21,22]. The core ions’ and valence electrons’ interaction were described by the projector augmented wave method (PAW), and the electron exchange-correlation part was described with the generalized gradient approximation (GGA) [23,24] by using the Perdew–Burke–Ernzerhof (PBE) approximation. In this work, the simulation calculations implemented here have been conducted on 2 × 2 × 2 supercell with 16 atoms in a body-centered cubic (bcc) structure [8]. Rare earth elements M (M = Y, La, Ce and Lu) were chosen to alloy in W with different concentrations with the form of W_1*−x*_M*_x_* (*x* = 0.0625, 0.1250, 0.1875, 0.2500 and 0.5000). The cutoff energy of 350 eV was chosen in our simulation calculations. The 11 × 11 × 11 Monkhorst–Pack scheme mesh for the geometry optimization of the supercell with 16 atoms and 13 × 13 × 13 mesh for systematic electronic calculation were used [15,25]. The total energy of each supercell was relaxed until the difference value was smaller than 10^−5^ eV. Each atom was completely relaxed until the energy was less than 10^−3^ eV/Å.

The components of the elastic tensor *C_ij_* without pressure were calculated by the computation of the stress-strain relationships [26]. Through Hooke’s law, we know that the stress-strain relationships can be expressed as

(*σ_i_*) = (*C_ij_*)(*ε_j_*). (1)

The response of the cubic crystal to the applied stress on the theoretical base of the continuum elasticity theory was well described by the three independent elastic constants, i.e., *C*_11_*, C*_12_ and *C*_44_ [27]. Therefore, the elastic constants for W_1−*x*_M*_x_* compounds can be calculated as follows:

*ε*^1^ = (0,0,0,*δ*,*δ*,*δ*),

*ε*^2^ = (*δ*,*δ*,0,*δ*,0,0),

*ε*^3^ = (*δ*,*δ*,*δ*,0,0,0),

According to the Voigt–Reuss–Hill scheme, the mechanical properties can be described from single crystal elastic constants [28,29]. The *G*, *B*, *E*, Poisson’s ratio (*ν*), and Cauchy pressure (*C*′) of W–M alloys were derived from *C*_11_, *C*_12_, and *C*_44_ via the following equations:

(2) $$B=\frac{C_{11}+2C_{12}}{3},$$

(3) $$G=\frac{C_{11}-C_{12}+3C_{44}}{5},$$

(4) $$E=\frac{9BG}{3B+G},$$

(5) $$\nu =\frac{E-2G}{2G},$$

(6) $$C^{\prime }=\frac{C_{12}-C_{44}}{2},$$

## 3. Results and Discussion

### 3.1. Mechanical Properties of BCC Crystal of Tungsten (W)

We first calculated the single-crystal elastic constants and mechanical properties of pure W. To determine the effect of the supercell size on the mechanical properties, we calculated the elastic constants of bcc W with different supercell size (the supercells with 16, 54 and 128 atoms), which are displayed in Table 1, together with other theoretical and experimental results. The results show that the elastic constants of W are almost unchanged with different supercell sizes, which well coincides with the previous literature. Therefore, the selected supercell with 16 atoms is reasonable [30].

The mechanical parameters, such as *B*, *G*, *E*, *B/G*, *ν* and *C*′ of pure W were calculated and are displayed in Table 2. For comparison, other existing theoretical and experimental data are also presented. The results basically agree well with the previously reported literature. Further, the calculated data were analyzed and compared, and it was found that the elastic constant *C_ij_* with previous results differed by a few percent. The error of the *B*, *G*, *E*, *ν* and *C*′ derived by *C_ij_* were within a reasonable range. The small discrepancies major came from the PBE approximation used in the calculations. In a word, our computational setup is reliable for calculating the mechanical properties of W alloying with rare earth elements.

### 3.2. Phase Stability and Electronic Properties

**Figure 1:**
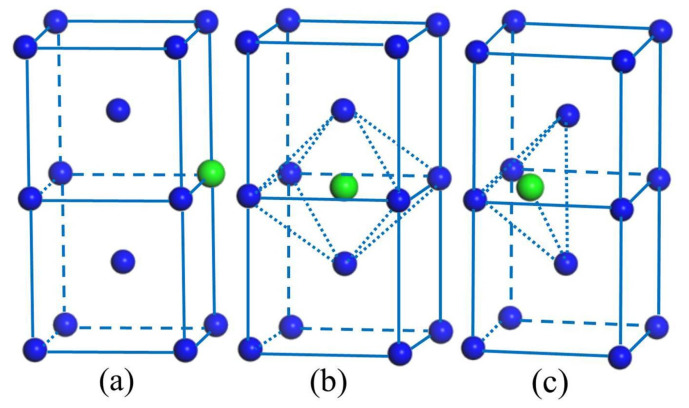
The substitutional site (**a**), the octahedral interstitial site (**b**) and the tetrahedral interstitial site (**c**) in bcc W structure. The large green spheres show the sites of the substitutional and interstitial; the blue spheres are the bcc W lattice sites.

The rare earth elements can occupy the substitutional site, the octahedral interstitial site (OIS) and the tetrahedral interstitial site (TIS) in body-centered cubic W metal, as shown in Figure 1a–c, respectively. The substitutional site has eight nearest neighbors located at 0.866a_0_ (a_0_ is the lattice constant of pure bcc W). The OIS has six nearest neighbors: two of them are located at 0.500a_0_ and four of them at 0.707a_0_. The TIS has four nearest neighbors at 0.559a_0_. As is evident, the free volume of the substitutional defect is largest and following are the octahedral defect and the tetrahedral defect.

To determine the relative stable positions of Y, La, Ce and Lu in W, the solution energies of the alloying elements with the substitutional site, TIS and OIS in W were calculated. The solution energy can be derived by Equation (7), when the M atom occupies the TIS or OIS [34].

(7) $$E_{M,\mathrm{interstitial}}^{\mathrm{sol}}=E_{NW,M}-E_{NW}-E_{M,\mathrm{isolated}}$$

where $E_{NW,M}$ represents the total energy of the system including all the atoms, i.e., *N* W atoms and a single M atom, $E_{NW}$ represents the total energy of the system including *N* W atoms and $E_{M,\mathrm{isolated}\;}$ represents the energy of an isolated M atom (a single M in system). The solution energy can be described by equation (8), when a W atom is substituted by an M atom [35].

(8) $$E_{M,\mathrm{substitutional}}^{\mathrm{sol}}=E_{\left(N-1\right)W,M}-E_{\left(N-1\right)W}-E_{M,\mathrm{isolated}\;}$$

Here $E_{\left(N-1\right)W,M}$ represents total energy of the system containing all the atoms, i.e., *N* − 1 W atoms and a single M atom, $E_{\left(N-1\right)W}$ depicts the total energy of the supercell comprising (*N* − 1) W atoms and $E_{M,\mathrm{isolated}\;}$ represents the energy of an isolated M atom (a single M in system). From the above definition, a negative solution energy means the alloying process is energetically favorable, and a more negative solution energy implies a more stable structure.

The 128 atom supercells with M atoms in W are used when the solution energy is calculated. The M atom solution energies in the supercell of W for the different sites are displayed in Table 3. The simulation calculations suggest that the solution energy is the minimum when the W atom is substituted by an M atom. Therefore, the M atom tends to occupy the substitution position within the bcc W. It is noted that there is a difference in solution energies between different rare earth elements at the same defect position. For substitutional alloying, the lowest solution energy corresponds to Lu (−6.49 eV), followed by Y, Ce and La. La has the highest solution energy (−3.55 eV). For TIS and OIS alloying, the lowest solution energy corresponds to Ce and the highest solution energy corresponds to La. Obviously, the solution energies are negative for all the M atoms in the substitutional site and positive for the M atoms in interstitial sites, indicating substitutional alloying is energetically favorable for all the four rare earth elements. It is also seen that the solution energies corresponding to the TIS are lower than those corresponding to OIS, indicating the TIS is more stable than the OIS.

**Figure 2:**
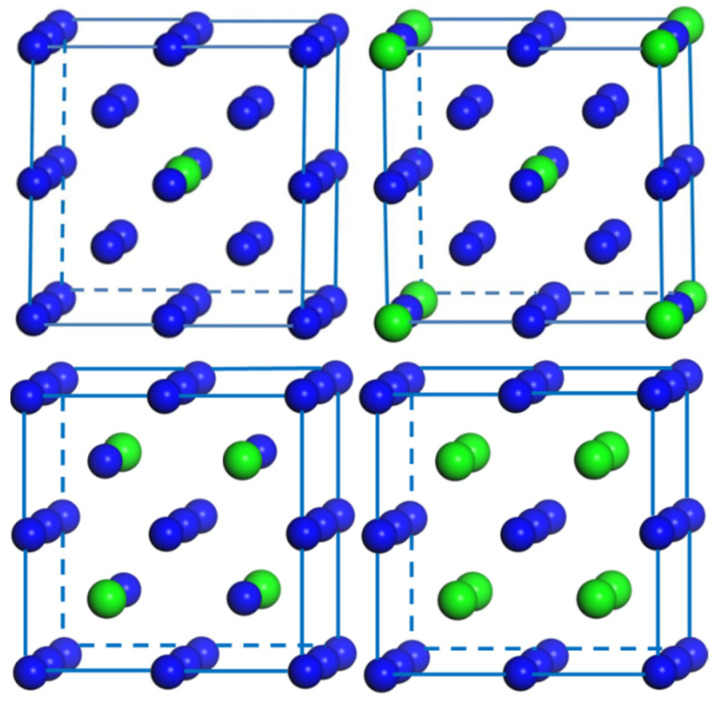
Schematic representation of energetically the most favorable atomic arrangements of the bcc W_1−*x*_M*_x_* (*x* = 0.0625, 0.125, 0.25 and 0.5) in a 2 × 2 × 2 supercell. Small blue and large green balls represent W and M (Y, La, Ce or Lu) atoms, respectively.

As stated above, the M atoms prefer to occupy the substitution position in W. With various concentrations, the rare earth element atomic sites and structures of W–M alloys should be determined. Since the configuration of the supercell with the energy minimization is relatively stable, the configurations with different alloying concentrations are established and adopted for the subsequent research. The model diagrams of the stable structures of the W_1−*x*_M*_x_* systems at different alloying contents are shown in Figure 2. Clearly, the W–M alloys possess bcc lattice, and the M atoms tend to sustain the structure with the highest symmetry.

**Figure 3:**
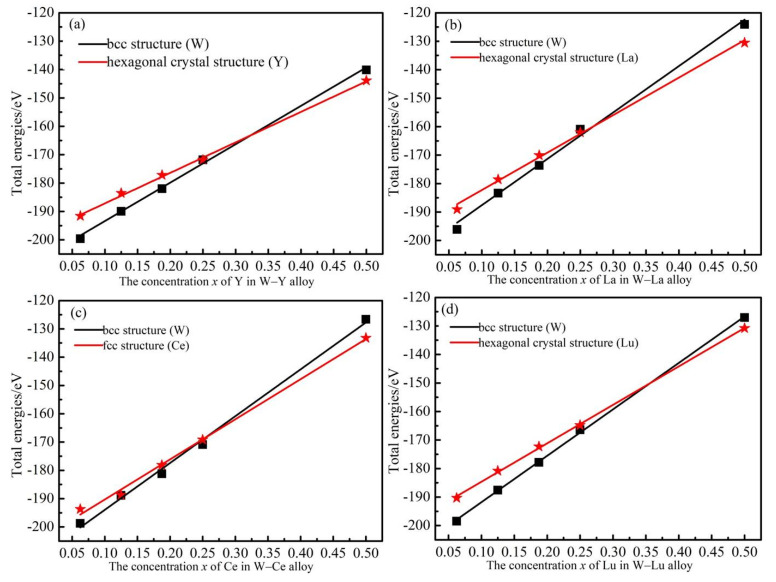
The total energy of the system as a function of different ingredients in bcc W and M structures. (**a**–**d**) represent W-Y, W-La, W-Ce and W-Lu systems, respectively.

We calculated the supercell energy of the alloying system based on the bcc lattice structure (pure W structure) and hexagonal lattice structure (pure rare earth element structure) and displayed them in Figure 3. The energies are basically linearly dependent on the content of the M concentrations for both lattice structures. The two fitting lines have an intersection point on each graph, which is the changing point of the stable structure by curve fitting method. The critical concentrations of the alloying elements *x* corresponding to the changing points are 0.32, 0.27, 0.25 and 0.36 for Y, La, Ce and Lu, respectively. For all the four alloying systems, the total energies in the hexagonal crystal structure are greater than those in the bcc structure when the alloying concentrations are less than the critical concentrations, indicating that the stable structure of the system is the bcc structure. When the alloying concentrations are larger than the critical concentrations, the total energies in the hexagonal crystal structure are lower than those in the bcc structure, meaning the stable structure of the system transforms to a hexagonal structure. To maintain the stability of the bcc structure of the W host, the calculations were done with the alloying concentrations limited to 0.25 hereafter.

**Figure 4:**
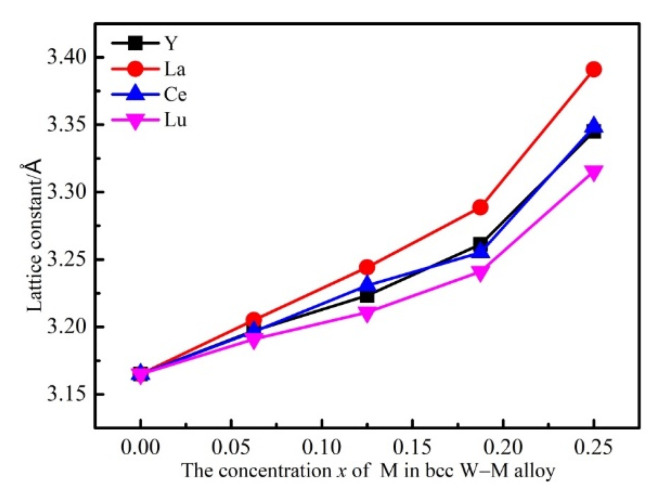
Equilibrium lattice constants of bcc W–M alloys as a function of M concentration.

The lattice constants of W–M alloys are displayed in Figure 4. It clearly describes the relationship between the equilibrium lattice constants of the system and the alloying element M concentration. It is shown that the lattice constants of the system increase with *x*, owing to the M atoms having greater atomic radii than W atoms. Combining Table 3 and Figure 4, it is found that the slope of curve was correlated with the solution energy of the single M atom in the substitution position, i.e., a smaller slope corresponds to more negative solution energy. For example, the slope of curve of the W–La line (red) is the largest and the solution energy of the La atom is the least negative. This can be intuitively interpreted as a stable structure accompanied with small lattice distortion. Following La are Ce, Y and Lu. As is known, the atomic radii of Y, La, Ce and Lu are 2.27 Å, 2.74 Å, 2.70 Å and 2.25 Å. The atomic radius (2.70 Å) of Ce is larger than the atomic radius (2.27 Å) of Y. However, the lattice constants of W–Y alloy are very similar to those of W–Ce alloy. The main factor is strong interaction between W and Ce atoms in W–Y alloys. In addition, it is noted that the slope of curve does not directly depend on the radius of the doped atoms. The lattice distortion can be explained by the electronic structures, which will be discussed in the following section.

### 3.3. Mechanical Properties

**Figure 5:**
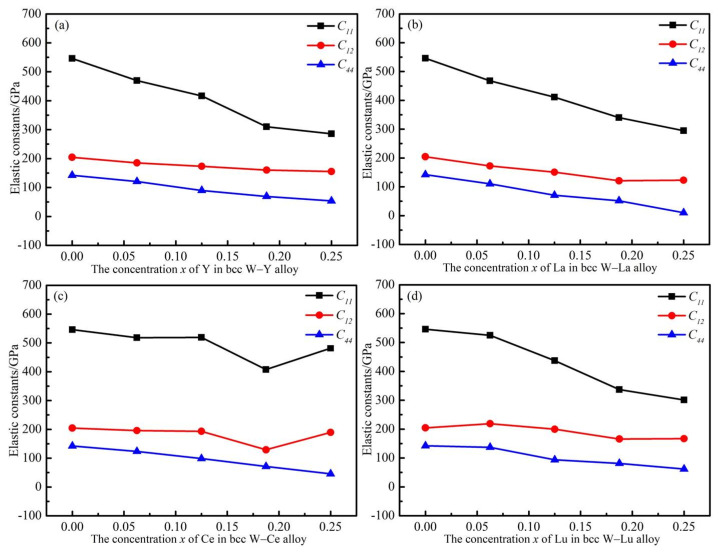
Three elastic constants of W–M binary alloys as functions of M concentration. (**a**–**d**) reresent W–Y, W–La, W–Ce, and W–Lu binary alloy system, respectively.

The relationship between the concentration of alloying elements M and the elastic properties of the W_1*−x*_M*_x_* solid solutions were systematically investigated, as displayed in Table 4 and Figure 5. The elastic constants of the pure W are also given for comparison. Following the Born–Huang elastic stability criteria for cubic crystals [36], the elastic constants should satisfy:

(9) $$C_{11}>0,\;\;C_{11}-C_{12}>0,\;\;C_{11}+2C_{12}>0,\;\;C_{44}>0$$

The first-principles calculation result clearly shows that the calculated elastic constants satisfy the above elastic stability condition. In all simulation calculations, the elastic stability of the bcc W_1*−x*_M*_x_* solid solution is guaranteed. Therefore, the binary alloy structure is ensured to be a mechanically stable model. As can be seen in Figure 5, all the *C*_11_ and *C*_44_ data explicitly show a regular decrease, and the *C*_12_ varies slightly and nonlinearly with the rare earth element content. Actually, the value of *C*_11_ represents the stiffness of cubic crystal. The black line (*C*_11_) decreases fastest in Figure 5a and decreases slowest in Figure 5c. The *C*_11_ value changes from 546.05 GPa for pure W to 285.60 GPa for W_0.766_Y_0.25_ and 294.55 GPa for W_0.766_La_0.25_ and 481.70 GPa for W_0.75_Ce_0.25_ and 301.07 GPa for W_0.75_Lu_0.25_ alloy. Consequently, the Y and La have the greater and the Ce has the least effect on *C*_11_ at the same alloying element concentration. The value of *C*_12_ reflects the ability of resistance to lateral deformation of the cubic crystals. In Figure 5 we can see that the *C*_12_ value does not change much when the M alloying element is added. Therefore, the main elastic constants of the systems decrease with alloy concentration, implying the mechanical strength of the systems become lower to some extent.

**Figure 6:**
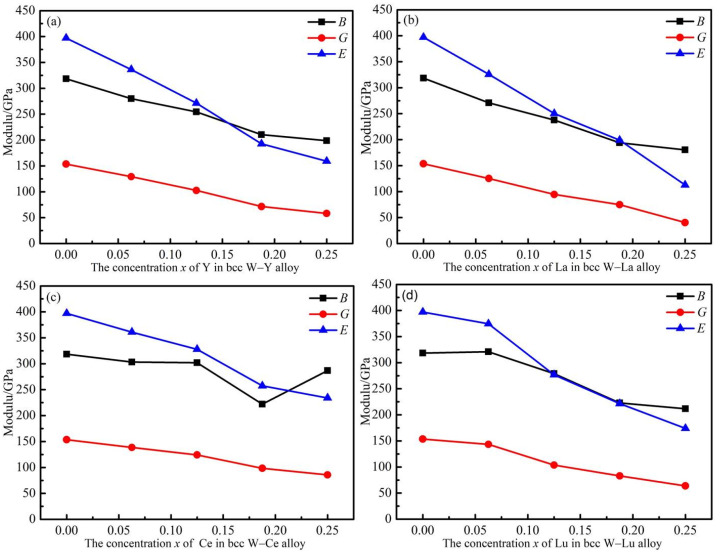
Bulk modulus (*B*), shear modulus (*G*) and Young’s modulus (*E*) of W–M binary alloys as functions of M concentration. (**a**–**d**) represent W–Y, W–La, W–Ce and W–Lu systems, respectively.

To further study the macroscopic mechanical properties of the W–M alloys, the derived *B*, *G*, *E*, *B/G*, *ν* and *C*′ were calculated with the above elastic constants as shown in Table 5 and Figure 6. As is indicated below, the calculated value (318.41 GPa) for the *B* of pure W basically agrees well with experimental results (314.3 GPa) [32]. The calculated value (198.79 GPa) for the *B* of W_0.75_Y_0.25_ alloy agrees well with previous reports (199.40 GPa). The calculated results for *E*, *G* and *C*′ of W_0.75_Y_0.25_ in previous studies are 177.4 GPa, 65.6 GPa and 45.00 GPa, respectively [7]. The data are very consistent with the results of our calculation. The elastic constants of W–La, W–Ce and W–Lu alloys are rarely studied, and there are almost no experimental data. As far as W–M alloys are concerned, the mechanical properties have a certain degree of comparability, when the concentrations for Y, La, Ce and Lu are the same. The method used in our calculation and derivation is exactly the same, and thus the resulting error is the same. Consequently, the elastic performance data of our calculation simulation is reliable. Obviously, it is found that all the physical quantities of *B*, *G* and *E* decrease with the increase of *x* concentration, meaning the mechanical strengths are somewhat reduced with the addition of the alloying elements. However, compared with Y, La and Lu, the decrement of the mechanical moduli of W–Ce alloys is smaller. For example, from the starting *B* of pure W (318.41 GPa), when the M concentration reaches 0.25, the *B* of W_0.75_M_0.25_ alloy slightly decreases to 286.87 GPa for Ce, and significantly to 198.79 GPa, 180.18 GPa and 211.68 GPa for Y, La and Lu, respectively. The same trend can also be obtained for *G* and *E*. So far, it can be concluded that Ce has stronger ability to maintain the mechanical strength of W when at the same alloying level compared to Y, La and Lu.

**Figure 7:**
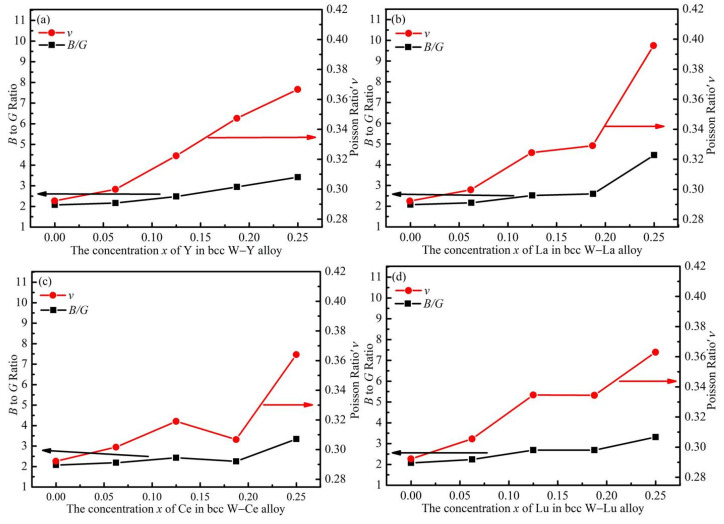
*B/G* ratio and Poisson’s ratio (*ν*) of W–M binary alloys as functions of M concentration. (**a**–**d**) represent W–Y, W–La, W–Ce and W–Lu systems, respectively.

We now turn to the ductility of the alloy materials. According to the Pugh criterion [36], the brittleness or ductility of the material can be determined by the ratio of the bulk over shear modulus *B/G*. The material is ductile when the *B/G* is higher than 1.75, otherwise it is considered brittle. The *ν* can be also used to judge the ductility of materials, i.e., the ductility of materials increases with the increase of the *ν* [37]. The *B/G* and *ν* values as functions of the Y, La, Ce and Lu concentration *x* are presented in Figure 7. As is shown, all *B/G* values are high than 1.75, implying that all the W alloys with Y, La, Ce and Lu are ductile materials in nature. It is known from the calculated data that all *B/G* values are larger than that of pure W (2.07) which suggests that we can increase the ductility of bcc W by alloying Y, La, Ce and Lu elements. With increasing M concentration, both the *B/G* and *ν* values increase, and hence the ductility improves.

It is noted that at the same alloying level, e.g., *x* = 0.25, the alloys with Y, Ce and Lu display essentially the same *B/G* and *ν* values. Combining the above fact that Ce has stronger ability to maintain the mechanical strength of W host than Y and Lu, we can conclude that Ce is a better alloying element to improving the ductility of W metal than Y and Lu. It is also noted that at the same alloying level, e.g., *x* = 0.25, W–La alloy displays greater *B*/*G* and ν values than Y, Ce and Lu alloys. From Figure 6 and Table 5, we can see that the mechanical moduli, e.g., bulk modulus B, of W–La alloy are very close to those of W–Y and W–Lu alloys. Therefore, from this point of view, we can say that La is a better alloying element to improving the ductility of W metal than Y and Lu.

In short, taking the two factors of mechanical moduli and ductility into consideration, La and Ce are better alloying elements than Y and Lu to improve the overall mechanical properties.

Qualitatively, the characteristic of atomic bonding can be determined by the *C*′. The brittle/ductile properties of metals relate to the characteristic of atomic bonding, and hence could be described by the *C*′ [37]. The materials are dominated by the metallic bonding interaction when the value of *C*′ is greater than zero. In other words, the bulk materials exhibit ductility. According to previous reports, the larger the value of *C*’, the stronger the metallicity of material and the better ductility [38,39]. For brittle materials, the *C*′ is generally less than zero. With more negative *C*′, the materials will be presenting more serious brittleness. As shown in Table 5, all the *C*′ values were greater than zero for the bulk W–M alloys, indicating the ductile nature of the materials. When the content of M increases in the W–M alloy system, the *C*′ value basically increases monotonically, which agrees well with the above discussed *B/G* and *ν*.

### 3.4. Electronic Structures

**Figure 8:**
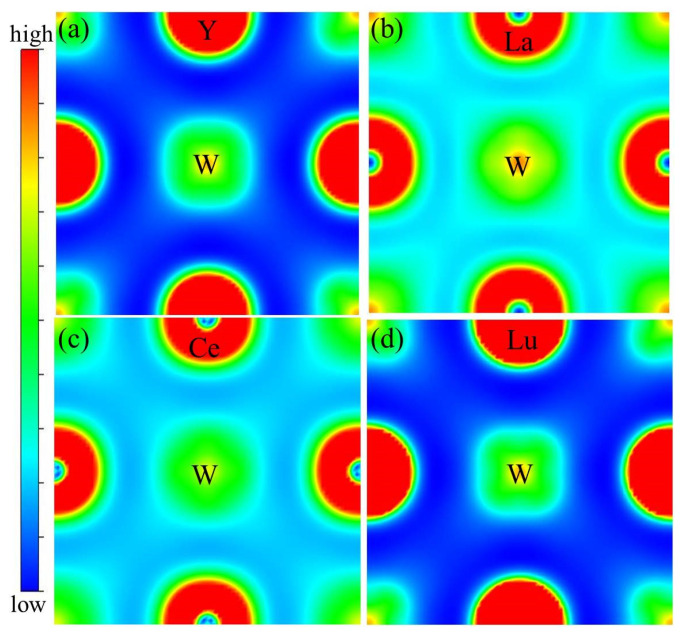
Charge density distribution for rare earth atoms incorporated in W. The (**a**–**d**) corresponds to Y, La, Ce and Lu located at the substitutional site.

To understand the mechanical properties of W_1*−x*_M*_x_* alloys, their charge density and electronic structures were analyzed. Figure 8 shows the charge density distributions corresponding to the Y–W, La–W, Ce–W and Lu–W alloys in the relaxed structures. It is seen that the highest charge density exists in the space between La and W atoms, illustrated in Figure 8b, showing maximum electronic interaction between the two elements. Following La–W are Ce–W, Y–W and Lu–W elements. As shown in Figure 8a,d, the charge density is very low, suggesting that the electronic interactions between W and Y or Lu are weak. Therefore, the strength of the interaction between M and W atoms plays an important role in the change of lattice constants, i.e., stronger interaction leads to larger lattice distortion. The charge density distribution well explains the lattice distortions of W–M alloys, as shown in Figure 4.

**Figure 9:**
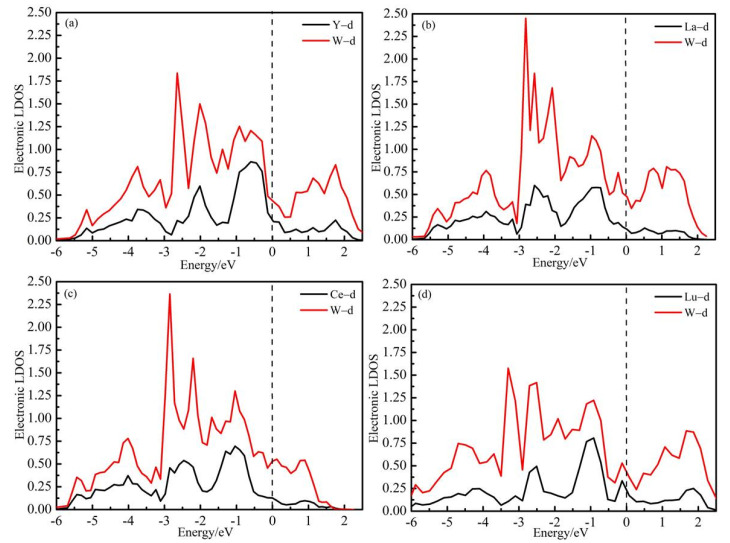
The local electronic densities of states (LDOS) of W and M atoms in bcc W_1*−x*_M*_x_* alloys, (**a**) W_0.875_Y_0.125_ alloy, (**b**) W_0.875_La_0.125_ alloy, (**c**) W_0.875_Ce_0.125_ alloy (**d**) and W_0.875_Lu_0.125_ alloy. The red lines are the LDOS of W nearest neighbors to M and black lines are the LDOS of M. The Fermi level was set as zero.

By calculating the density of states (DOS) of the W_1*−x*_M*_x_* alloys, the bonding interaction can be understood, and the mechanical properties and structural stability mechanism can be revealed. The electronic structures of W_0.875_M_0.125_ alloys were analyzed as an example. Figure 9 shows the calculated local electronic densities of states (LDOS) of W and M atoms in bcc W_0.875_M_0.125_ alloys. The red lines are the LDOS of W nearest neighbors to M and black lines are the LDOS of M with *d*-state. It can be seen from Figure 9 that there is no energy gap at Fermi level for W_0.875_M_0.125_ alloys, meaning that all these compounds present a metallic character. When M is doped into the W lattice, as shown in Figure 9, we note that the shapes of d-states’ LDOS of M atoms are similar to the shapes of d-states’ LDOS of W atoms, reflecting the strong hybridization between these states. It can also be found that the hybridization of W_0.875_La_0.125_ and W_0.875_Ce_0.125_ are obviously higher than W_0.875_Y_0.125_ and W_0.875_Lu_0.125_ alloys, which indicates that the bond interactions are the stronger in W_0.875_La_0.125_ and W_0.875_Ce_0.125_ alloys, which agrees well with the lattice distortions of W–M alloys shown in Figure 4.

**Figure 10:**
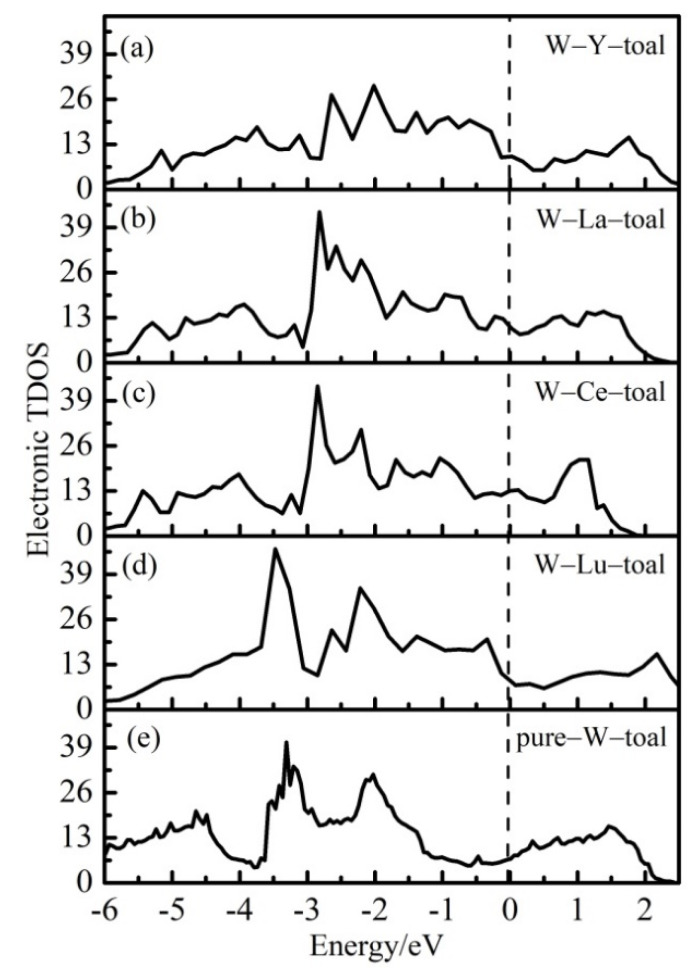
The total densities of states (TDOS) of bcc W_1−*x*_M*_x_* alloys and pure W, (**a**) W_0.875_Y_0.125_ alloy, (**b**) W_0.875_La_0.125_ alloy, (**c**) W_0.875_Ce_0.125_ alloy, (**d**) W_0.875_Lu_0.125_ alloy (**e**) and pure W.

To obtain further insight into the relationship between electronic structures and mechanical properties of W_0.875_M_0.125_ alloys, the total densities of states (TDOS) of bcc W_1−*x*_M*_x_* alloys and pure W were calculated and are displayed in Figure 10. As is known, the density of states at the Fermi level can indirectly reflect the hardness of the alloy, and the smaller the value, the greater the hardness [40]. It is seen that the main peaks of W_0.875_M_0.125_ alloys move to the right relative to that of pure W, and hence leading to greater TDOS at the Fermi level, i.e., the bonding in the W_1−*x*_M*_x_* alloys becomes more metallic than that in pure W, and the hardness of the alloys is getting lower and the ductility is improved. Specifically, the TDOS at the Fermi level of W_0.875_La_0.125_ and W_0.875_Ce_0.125_ are higher than those of W_0.875_Y_0.125_ and W_0.875_Lu_0.125_ alloys, which indicates that the bond interactions in W_0.875_La_0.125_ and W_0.875_Ce_0.125_ alloys are more metallic than the other two. Therefore, La and Ce are better alloying elements than Y and Lu to improve ductility. This is consistent with the above analysis of mechanical moduli and ductility.

## 4. Conclusions

To summarize, the effects of Y, La, Ce or Lu alloying on lattice stability and mechanical properties of W_1*−x*_M*_x_* have been studied by first principles calculations. The lattice constants, solution energies and elastic constants of W_1*−x*_M*_x_* (*x* = 0.0625, 0.125, 0.1875, 0.25) alloys were calculated by the 16-atom solid solution model and the M concentration effects on the fundamental stability and performance of the W–M alloys were specifically addressed. The Y, La, Ce and Lu atoms tend to occupy the substitution position in W. The bcc W–M alloy structure was stable when the Y, La, Ce or Lu concentration *x* was less than 0.32, 0.27, 0.25 or 0.37 respectively. The substitutional solid solution of W–M binary can be formed on the atomic scale. The lattice constant increased linearly with the increase of Y, La, Ce or Lu alloying content. Furthermore, the elastic constants and moduli of W–Y, W–La and W–Lu alloys were found to decrease somewhat with an increasing concentration of *x*, implying the mechanical strengths of W–M alloys become lower than that of pure W. However, we demonstrated that all the W–Y, W–La, W–Ce and W–Lu alloys are ductile materials by calculating the *B/G* ratio, the Poisson’s ratio *ν* and Cauchy pressure *C*′, and increasing the rare earth element concentration was helpful in improving the ductility. Considering both factors of mechanical strength and ductility, La and Ce are better alloying elements than Y and Lu to improve the overall mechanical properties.

## Figures and Tables

**Table 1:** Elastic constants of pure W.

Method	Configuration	*C*_11_/GPa	*C*_12_/GPa	*C*_44_/GPa
Present work	16 atom cell	546.05	204.59	142.22
Present work	54 atom cell	545.50	210.96	143.69
Present work	128 atom cell	558.42	205.48	143.58
Theory [31]	54 atom cell	529.94	211.19	140.59
Experiment [32]	-	533.0	205.0	163.0
Theory [32,33]	-	533.0	207.0	163.0

**Table 2:** Bulk modulus (*B*), shear modulus (*G*), Young’s modulus (*E*), *B/G* ratio, Poisson’s ratio (*ν*) and Cauchy pressure (*C*′) of pure W.

Method	*B*/GPa	*G*/GPa	*E*/GPa	*B/G*	*ν*	*C*′
Present work	318.41	153.62	397.02	2.07	0.29	31.19
Theory [31]	317.44	148.11	384.52	2.14	0.30	35.29
Experiment [32]	314.33	163.40	417.80	1.92	0.28	21.00
Theory [32,33]	322.33	173.00	440.24	1.86	0.27	22.00

**Table 3:** Solution energies of single M atom in bcc tungsten for different sites calculated in VASP.

Elements	$E_{\mathrm{sub}}^{\mathrm{sol}}/\mathrm{eV}$	$E_{\mathrm{tetra}}^{\mathrm{sol}}/eV$	$E_{\mathrm{octa}}^{\mathrm{sol}}/eV$
Y	−5.58	9.76	11.00
La	−3.55	10.22	12.32
Ce	−5.26	7.78	10.03
Lu	−6.49	9.27	10.28

**Table 4:** Elastic constants of bcc W_1−*x*_M*_x_*.

Composition	Element	*C*_11_/GPa	*C*_12_/GPa	*C*_44_/GPa
Pure W	W	546.05	204.59	142.22
W_0.9375_M_0.0625_	Y	469.35	185.26	120.80
	La	467.89	172.44	110.29
	Ce	518.31	195.78	123.57
	Lu	524.88	218.88	137.08
W_0.875_M_0.125_	Y	416.58	173.34	89.89
	La	410.96	150.97	70.84
	Ce	519.02	193.33	98.54
	Lu	437.15	199.74	93.66
W_0.8125_M_0.1875_	Y	310.02	160.41	69.25
	La	340.18	121.00	51.72
	Ce	407.71	129.16	71.29
	Lu	336.87	165.90	81.33
W_0.75_M_0.25_	Y	285.60	155.39	53.60
	La	294.55	123.00	10.10
	Ce	481.70	189.46	45.56
	Lu	301.07	166.98	61.69

**Table 5:** Bulk modulus (*B*), shear modulus (*G*), Young’s modulus (*E*), *B/G* ratio, Poisson’s ratio(*ν*) and Cauchy pressure (*C*′) for bcc W_1*−x*_M*_x_* alloys.

Composition	Element	*B*/GPa	*G*/GPa	*E*/GPa	*B/G*	*ν*	*C*′/GPa
Pure W	W	318.41	153.62	397.02	2.07	0.29	31.19
W_0.9375_M_0.0625_	Y	279.96	129.30	336.14	2.17	0.30	32.23
	La	270.92	125.26	325.61	2.16	0.30	31.07
	Ce	303.29	138.65	360.94	2.19	0.30	36.10
	Lu	320.88	143.45	374.53	2.24	0.31	40.90
W_0.875_M_0.125_	Y	254.42	102.58	271.29	2.48	0.32	41.73
	La	237.63	94.50	250.32	2.51	0.32	40.07
	Ce	301.89	124.26	327.81	2.43	0.32	47.40
	Lu	278.88	103.68	276.74	2.69	0.33	53.04
W_0.8125_M_0.1875_	Y	210.28	71.47	192.60	2.94	0.34	45.58
	La	194.06	74.87	199.01	2.59	0.33	34.64
	Ce	222.01	98.48	257.39	2.25	0.31	28.94
	Lu	222.89	82.99	221.49	2.69	0.33	42.29
W_0.75_M_0.25_	Y	198.79	58.21	159.08	3.42	0.37	50.90
	La	180.18	40.37	112.69	4.46	0.40	56.45
	Ce	286.87	85.78	234.03	3.34	0.36	71.95
	Lu	211.68	63.83	174.00	3.32	0.36	52.65

## Data Availability

The data presented in this study are available on request from the corresponding author.
